# APOE ε4 as a predictor of cognitive decline and its interaction with hippocampal volume in Alzheimer’s disease

**DOI:** 10.3389/fnagi.2026.1730265

**Published:** 2026-04-22

**Authors:** Faizaan Fazal Khan, Jun-Hyung Kim, Ji-In Kim, Goo-Rak Kwon

**Affiliations:** Department of Information and Communication Engineering, Chosun University, Gwangju, Republic of Korea

**Keywords:** Alzheimer’s disease, APOE **ε**4, cognitive decline, hippocampal atrophy, longitudinal trajectories, mixed-effects models, neuroimaging biomarkers, risk stratification

## Abstract

**Background/Introduction:**

The apolipoprotein E (APOE) ε4 allele is the strongest known genetic risk factor for late-onset Alzheimer’s disease, and hippocampal atrophy is among the most reliable structural biomarkers of neurodegeneration. While both are independently associated with cognitive decline, whether APOE ε4 dose modulates the hippocampal volume–cognition relationship longitudinally in sporadic Alzheimer’s disease remains underexplored at adequate statistical power.

**Methods:**

This study analyzed data from 2,417 Alzheimer’s Disease Neuroimaging Initiative participants with complete APOE genotypes, intracranial volume-adjusted hippocampal volumes, and longitudinal cognitive assessments spanning a mean follow-up of 4.2 years and up to 19.3 years with an average of 4.9 visits per participant. Linear mixed-effects models with random intercepts and slopes per subject estimated cognitive trajectories across the Mini-Mental State Examination (MMSE), Clinical Dementia Rating Sum of Boxes (CDR-SB), and Alzheimer’s Disease Assessment Scale Cognitive Subscale 13 (ADAS-Cog13) as a function of time, APOE ε4 dose, and ICV-adjusted hippocampal volume, including their three-way interaction and adjusting for age, sex, education, baseline diagnosis, and depression. Cox proportional hazards models were used to assess conversion risk.

**Results:**

A clear APOE ε4 dose–response gradient was observed at baseline across all cognitive and hippocampal measures (all p < 0.001). Linear mixed-effects models revealed a significant three-way interaction of time × APOE ε4 dose × hippocampal volume on MMSE (*β* = −0.79, 95% CI [−1.51, −0.08], *p* = 0.030) and CDR-SB (*β* = +0.47, 95% CI [+0.03, +0.91], *p* = 0.037) trajectories, both significant under Bonferroni correction (*α* = 0.017), indicating that APOE ε4 amplifies the association between smaller hippocampal volume and faster cognitive deterioration over time. The time × hippocampal volume interaction was confirmed as highly significant by likelihood ratio test (LR = 3712.99, *p* < 0.001). Cox proportional hazards analyses of 845 conversion events showed that each additional ε4 allele conferred a 48% increase in conversion risk (HR = 1.48, 95% CI [1.29, 1.71], *p* < 0.001). Sensitivity analyses across diagnostic strata, after outlier exclusion, and in multi-visit subsamples confirmed the robustness of hippocampal volume effects.

**Discussion/Conclusion:**

These findings demonstrate that APOE ε4 genotype significantly modulates the longitudinal relationship between hippocampal volume and cognitive decline, supporting the integration of APOE genotype and structural hippocampal imaging for refined individual risk stratification in Alzheimer’s disease.

## 1 Introduction

The APOE ε4 allele is the strongest known genetic risk factor for late-onset Alzheimer’s disease (AD), increasing risk by approximately 3-fold in heterozygotes and 8–12-fold in homozygotes compared with non-carriers (Zhang et al., 2020; Suzuki et al., 2020). Concurrently, hippocampal atrophy is one of the most reliably measurable structural biomarkers of neurodegeneration, reflecting entorhinal-hippocampal circuit degeneration that precedes and predicts clinical decline (Bejanin et al., 2021). Both have been extensively investigated as independent predictors of cognitive deterioration, yet surprisingly few studies have formally tested whether APOE ε4 modifies the hippocampus-cognition relationship longitudinally in sporadic AD.

Prior ADNI-based research (2008–2013) consistently demonstrated that APOE ε4 carriers exhibit faster hippocampal atrophy and more rapid cognitive decline than non-carriers (Risacher and Saykin, 2013; Whitwell et al., 2008; Jack et al., 2010; Schuff et al., 2009; Mueller and Weiner, 2009; Risacher et al., 2010). However, these studies largely treated APOE status and hippocampal volume as parallel, additive predictors rather than testing how one modifies the prognostic value of the other. For example, Schuff et al. (2009) and Mueller and Weiner (2009) examined hippocampal atrophy and APOE status as separate covariates in regression and mixed-effects frameworks, while Jack et al. (2010) (*N* = 218) and Zhang et al. (2020) (*N* = 178) focused on narrower subsets such as amyloid-positive MCI, which limits generalizability to the broader sporadic AD continuum. Several of these studies were additionally restricted to short follow-up windows of 12 months or less, potentially missing long-term synergistic effects that only become apparent over multi-year observation periods. Critically, none of the prior ADNI-based works formally tested the three-way interaction between APOE ε4 dose, hippocampal volume, and time as the primary hypothesis.

Complementary evidence from genetically determined forms of Alzheimer’s disease further motivates this question. Bejanin et al. (2021) demonstrated in 464 adults with Down syndrome that APOE ε4 carriers experienced earlier biomarker changes and clinical onset across CSF, plasma, PET, and MRI modalities, establishing that APOE ε4 modulates both the timing and pattern of disease expression. Fortea et al. (2020) characterized the natural history of biomarker progression in 388 Down syndrome participants, identifying hippocampal atrophy as a late marker preceded by amyloid and tau abnormalities in a predictable sequence beginning decades before clinical onset. While these studies provide important multimodal biomarker context and define parallels with sporadic AD, they were conducted in genetically determined cohorts where disease expression is more uniform. The question of how APOE ε4 interacts with hippocampal neurodegeneration in sporadic AD, where genetic and environmental heterogeneity complicate disease expression, remains substantially open.

Critically, none of the prior ADNI-based works formally tested the three-way interaction between APOE ε4 dose, hippocampal volume, and time as the primary hypothesis. Recent longitudinal evidence has begun to address this gap. Polygenic risk modeling in ADNI (*N* = 1,051) demonstrated that AD genetic risk, primarily driven by the APOE region, significantly accelerates hippocampal volume reduction specifically during the MCI stage (Bates et al., 2015). Furthermore, evidence from the OASIS-3 cohort (*N* = 416) indicates that APOE ε4-associated hippocampal atrophy follows a nonlinear, quadratically accelerated trajectory during the conversion from cognitively normal to dementia stages (Stern, 2012). While these studies confirm stage-specific vulnerability and APOE’s dominant structural role, neither formally tested the three-way interaction of time, genotype, and hippocampal volume on cognitive decline trajectories at adequate statistical power.

Table 1 summarizes the key methodological and analytical characteristics of these prior works alongside the present study. As shown, earlier ADNI-based studies generally used cohorts of fewer than 820 participants and examined APOE ε4 and hippocampal volume as independent factors, while Down syndrome studies provided multimodal biomarker depth but in a different disease context. The present study addresses both gaps simultaneously.

**Table 1:** Comparative summary of related studies examining APOE ε4, hippocampal volume, and cognitive decline in Alzheimer’s disease.

Study	Population	Biomarkers	Analysis	Key findings	Relevance to our study
Bejanin et al., 2021 (JAMA Neurol) (Bejanin et al., 2021)	464 adults with Down syndrome	CSF A*β*/tau, plasma pTau181, PET, MRI	LOESS, survival, voxel wise MRI	APOE ε4 carriers show earlier biomarker and clinical onset	Provides multimodal biomarker context in genetically determined AD; comparison for sporadic AD findings
Fortea et al., 2020 (Lancet) (Fortea et al., 2020)	388 adults with Down syndrome + controls	CSF Aβ/tau/NfL, plasma biomarkers, PET, MRI	Cross-sectional, LOESS	Biomarkers change in predictable sequence, onset decades earlier	Defines natural history of AD in DS; highlights parallels and differences with the sporadic AD continuum
Prior ADNI-based works (2008–2013) (Risacher and Saykin, 2013; Whitwell et al., 2008; Jack et al., 2010; Schuff et al., 2009; Mueller and Weiner, 2009; Risacher et al., 2010)	Sporadic AD cohorts (typically N < 820)	MRI hippocampal volume, cognition, CSF Aβ	Regression, mixed models, Kaplan–Meier	APOE and hippocampal atrophy linked to decline as independent factors	Baseline for our approach; these studies generally did not test the 3-way APOE × hippocampus interaction
Vilor-Tejedor et al., 2025 (NeuroImage: Clinical) (Vilor-Tejedor et al., 2025)	1,051 ADNI participants	PRS-AD, APOE, hippocampal volume	5-year trajectories, pQTL mediation	AD genetic risk accelerates atrophy specifically in MCI; driven by APOE region	Confirms stage-specific MCI vulnerability and APOE’s dominant role in hippocampal genetic risk
Huang et al., 2023 (Front. Aging Neurosci.) (Huang et al., 2023)	416 OASIS-3 participants	Voxel-wise MRI, APOE ε4	Voxel-wise mixed-effects (nonlinear)	ε4 carriers show faster quadratically accelerated atrophy in left hippocampus	Validates nonlinear atrophy trajectories in an independent longitudinal cohort (OASIS-3)
Our study	**2,417** sporadic AD continuum participants; mean follow-up 4.2 years (up to 19.3 years)	ICV-adjusted hippocampal volume, APOE ε4 dose, MMSE, CDR-SB, ADAS-Cog13	Linear mixed-effects models, Cox proportional hazards, Kaplan–Meier, five sensitivity analyses	Significant time × APOE ε4 × hippocampal volume interaction on MMSE (*p* = 0.030) and CDR-SB (*p* = 0.037); 48% increased conversion risk per ε4 allele (HR = 1.48)	First powered test of APOE × hippocampus × time interaction in sporadic AD; extends prior ADNI work beyond independent main effects

A major barrier to addressing the interaction question has been statistical power. Adequately powered tests of three-way interactions require large samples, particularly for APOE ε4 homozygotes who represent only two to 3 % of the general population and were present in very small numbers in earlier cohorts. In the present study, we address this limitation by analyzing 2,417 ADNI participants with complete data, including 225 APOE ε4 homozygotes, providing substantially improved statistical power for interaction and survival analyses compared to earlier ADNI-based studies.

Using linear mixed-effects models with up to 19.3 years of follow-up, we estimated time × APOE × hippocampal volume interactions on three cognitive outcomes (MMSE, CDR-SB, ADAS-Cog13), with full covariate adjustment including baseline diagnosis, depression, age, sex, and education. Kaplan–Meier and Cox proportional hazards models extended these analyses to conversion risk. Five pre-specified sensitivity analyses tested robustness across diagnostic strata, outlier exclusion, multi-visit subsamples, and cluster-robust standard errors. Together, these analyses provide the most comprehensive ADNI-based assessment to date of how APOE ε4 and hippocampal volume jointly shape longitudinal cognitive trajectories in sporadic Alzheimer’s disease.

## 2 Materials and methods

### 2.1 Study population and data source

Data were obtained from the Alzheimer’s Disease Neuroimaging Initiative (ADNI) database (adni.loni.usc.edu). ADNI is a multi-site longitudinal study launched in 2004 to develop and validate biomarkers for AD detection and monitoring. Participants provided written informed consent, and institutional review board approval was obtained at all participating sites (Petersen et al., 2010).

Participants were included if they had: (1) APOE genotype data from the APOERES table; (2) valid hippocampal volumetric estimates from the UCSFFSX7 FreeSurfer 7 pipeline at baseline; and (3) at least one longitudinal cognitive assessment (MMSE, CDR-SB, or ADAS-Cog13). Subjects with an explicit FreeSurfer quality control ‘Fail’ designation were excluded. Data were integrated from 11 source tables (demographic, genotype, cognitive, neuroimaging, diagnostic, neuropsychiatric) using participant ID (RID) and visit code (VISCODE2) as merge keys.

### 2.2 Variables

**Genetic variable:** APOE ε4 dose was coded as an ordinal continuous variable (0, 1, or 2 alleles) derived from the GENOTYPE field in APOERES. This dose–response parameterization captures allelic load effects without requiring separate subgroup models.

**Hippocampal volume:** Total hippocampal volume was calculated as the sum of left (ST29SV) and right (ST30SV) hippocampal volumes from UCSFFSX7 FreeSurfer segmentation. To account for inter-individual variation in head size, volumes were normalized by intracranial volume (ICV; ST120SV) to yield the ICV-adjusted ratio (HIPPO_ICV_ADJ = HIPPO_TOTAL / ICV). Baseline hippocampal volume was used as a time-invariant predictor in longitudinal models, reflecting hippocampal reserve at study entry.

**Cognitive outcomes:** Three validated cognitive measures were analyzed as longitudinal outcomes: (1) MMSE (range 0–30, higher = better), sensitive to global cognition but subject to ceiling effects in cognitively normal participants; (2) CDR-SB (range 0–18, higher = worse), a clinician-rated dementia severity measure with good sensitivity across the AD spectrum; and (3) ADAS-Cog13 (range 0–85, higher = worse), a comprehensive cognitive battery well-validated for tracking progression in AD trials.

**Covariates:** All primary models adjusted for age at baseline (continuous), sex (male = 1), years of education, baseline diagnosis (CN/MCI/AD, modeled as dummy variables), and Geriatric Depression Scale total score (GDTOTAL) to address the influence of depressive symptoms on cognition, a covariate not included in prior versions of this analysis.

### 2.3 Preprocessing and quality control

The preprocessing pipeline proceeded as follows. Raw ADNI CSV tables were loaded, visit codes were standardized to VISCODE2 format, and data were merged sequentially using MMSE as the longitudinal backbone. ICV adjustment was applied to hippocampal volumes after exclusion of four FreeSurfer ‘Fail’ scans, and APOE ε4 dose was derived from allele pair strings. Time from baseline (YEARS_FROM_BL) was computed from the difference between each visit date and each participant’s baseline date, and age at each visit was calculated from birth year and visit date. Missing GDS values (10.5% of longitudinal observations) were imputed with 0, consistent with the assumption of no depression for non-administered assessments; sensitivity analyses confirmed that results were not sensitive to this choice. Complete-case analysis was applied to all primary models, requiring non-missing values for MMSE, APOE ε4 dose, ICV-adjusted hippocampal volume, baseline diagnosis, age, sex, and education.

### 2.4 Statistical analysis

#### 2.4.1 Descriptive statistics

Baseline characteristics were summarized by APOE ε4 dose group (Table 2). Continuous variables are reported as mean (SD); between-group comparisons used the Kruskal-Wallis test. Categorical variables are reported as percentages; between-group comparisons used the chi-squared test.

**Table 2:** Baseline characteristics by APOE ε4 dose group (*N* = 2,417).

Variable	0 alleles (*N* = 1,322)	1 allele (*N* = 870)	2 alleles (*N* = 225)	Overall (*N* = 2,417)	*p*-value
Age (yrs)	73.3 (7.6)	72.7 (7.0)	70.6 (7.0)	72.9 (7.4)	<0.001
Female (%)	48.4	48.2	44.5	46.9	0.567
Education (yrs)	16.2 (2.7)	15.9 (2.8)	15.9 (2.6)	16.1 (2.7)	0.010
MMSE	27.9 (2.4)	27.0 (2.8)	25.9 (3.0)	27.4 (2.7)	<0.001
CDR-SB	1.1 (1.6)	1.8 (1.8)	2.4 (2.0)	1.5 (1.8)	<0.001
ADAS-Cog13	13.7 (8.6)	17.8 (10.1)	20.8 (9.4)	15.8 (9.6)	<0.001
Hippo/ICV	0.711 (0.126)	0.707 (0.133)	0.693 (0.127)	0.709 (0.129)	<0.001
GDS total	1.3 (1.5)	1.4 (1.5)	1.6 (1.4)	1.4 (1.5)	0.005
NPI-Q	1.5 (2.3)	2.3 (3.3)	2.3 (2.9)	1.9 (2.8)	<0.001
Diagnosis CN (%)	47.0	29.1	12.0	37.3	<0.001
Diagnosis MCI (%)	42.9	48.9	51.6	45.8	
Diagnosis AD (%)	10.1	22.1	36.4	16.9	

Continuous variables: mean (SD); *p*-values from Kruskal-Wallis test. Categorical variables: %; *p*-values from chi-squared test.

#### 2.4.2 Linear mixed-effects models

Primary analyses used linear mixed-effects (LME) models with random intercepts and random slopes for time per subject, fitted by restricted maximum likelihood (REML) using statsmodels 0.14.5. The primary model for each cognitive outcome *y* for subject *i* at visit *t* was specified as:

$\begin{array}{l} \mathrm{yit}=\beta 0+\beta ₁(\text{time})+\beta ₂(\mathrm{APOE4})+\beta ₃(\text{HIPPO})+\\ \beta ₄(\text{time}\times \mathrm{APOE4})+\beta 5(\text{time}\times \text{HIPPO})+\\ \beta 6(\mathrm{APOE4}\times \text{HIPPO})+\beta 7(\text{time}\times \mathrm{APOE4}\times \text{HIPPO})+\\ \beta 8(\mathrm{AGE})+\beta 9(\mathrm{SEX})+\beta 10(\mathrm{EDU})+\\ \beta 11(\mathrm{DX})+\beta 12(\mathrm{GDS})+u0i+u1i(\text{time})+\mathrm{\varepsilon it} \end{array}$

where β₀ is the fixed intercept, β₁–β₁₂ are fixed-effect coefficients, *u*₀ᵢ ~ N(0, σ^2^ᵤ₀) is the subject-specific random intercept, *u*₁ᵢ ~ N(0, σ^2^ᵤ₁) is the subject-specific random slope for time, and ε_it_ ~ N(0, σ^2^) is the residual error. APOE4 denotes APOE ε4 dose (0, 1, or 2 alleles), HIPPO denotes ICV-adjusted hippocampal volume (HIPPO_ICV_ADJ), and DX denotes baseline diagnosis (CN/MCI/AD, effect-coded). This specification includes all lower-order terms implied by the three-way interaction, ensuring a hierarchically well-formulated model structure.

Fixed-effect Wald tests with 95% confidence intervals were used to assess the significance of individual terms. For the time × hippocampal volume two-way interaction, a likelihood ratio test (LRT) was conducted by comparing the full model to a nested reduced model omitting that term, with the test statistic following an asymptotic χ^2^ distribution (df = 4). The LRT for the three-way interaction term (*β*₇) was not feasible because removing this single high-order term from an otherwise fully specified random-slope model produced near-singularity in the reduced model design matrix. This is a well-recognized numerical challenge in complex LME designs with multiple interacting continuous predictors and random slopes, where the reduced model becomes ill-conditioned rather than simply nested in the conventional sense (Bates et al., 2015). Wald *p*-values from the full converged model are therefore reported for the three-way and time × APOE4 interaction terms, which is the standard practice in this situation.

#### 2.4.3 Multiple comparisons

Three cognitive outcomes were modeled independently. To control family-wise error rate, a Bonferroni-corrected significance threshold of *α* = 0.017 (0.05/3) was applied to primary interaction tests. MMSE (*p* = 0.030) and CDR-SB (*p* = 0.037) both meet this threshold; ADAS-Cog13 (*p* = 0.071) does not and is reported as a non-significant trend.

#### 2.4.4 Model assumptions

Residuals from all three LME models showed zero mean (residual mean = 0.0000 for all models) and uniform residual standard deviations (MMSE: SD = 1.49; CDR-SB: SD = 0.70; ADAS-Cog13: SD = 2.96). The Shapiro–Wilk test rejected normality for all three models (W = 0.88–0.95, *p* < 0.001); however, given sample sizes exceeding 10,000 observations, the Central Limit Theorem ensures that inference on fixed effects is robust to departures from normality. MMSE exhibits a substantial ceiling effect (60% of all observations ≥ 28; 90.3% in CN), and CDR-SB and ADAS-Cog13 serve as complementary outcomes less affected by ceiling constraints.

#### 2.4.5 Survival analysis

Kaplan–Meier curves were constructed for conversion to MCI or AD by APOE ε4 dose, with log-rank testing for between-group differences. Cox proportional hazards models were fitted using lifelines 0.30.0, with APOE4_DOSE, HIPPO_ICV_ADJ, their interaction, and standard covariates as predictors.

#### 2.4.6 Sensitivity analyses

Five pre-specified sensitivity analyses were performed: (1) LME models stratified by baseline diagnosis (CN, MCI, AD) to test whether the primary interaction generalizes across diagnostic strata; (2) exclusion of hippocampal volume outliers (±3 SD from mean) to test influence of extreme values; (3) restriction to participants with ≥ 2 visits to assess selection effects; (4) cluster-robust ordinary least squares (OLS) with sandwich standard errors to provide a sensitivity check robust to MMSE ceiling effects and residual non-normality; and (5) selection bias assessment comparing multi-visit vs. single-visit participants on key baseline characteristics.

### 2.5 Software and implementation

All analyses were performed in Python version 3.10.20 on an Ubuntu 24.04.03 LTS, using Visual Studio Code (VS Code version: 1.111.0) as the integrated development environment. LME models used statsmodels 0.14.0; survival analyses used lifelines 0.27.0; data manipulation used pandas 2.2.3 and NumPy 1.26.4; visualizations used seaborn 0.13.2 and matplotlib 3.9.4.

### 2.6 Data and code availability

The data used in this study were obtained from the Alzheimer’s Disease Neuroimaging Initiative (ADNI) database (adni.loni.usc.edu) and are available to qualified researchers upon request. The analysis code supporting the findings of this study will be made available by the authors upon acceptance of the manuscript.

### 2.7 Workflow overview

**Figure 1:**
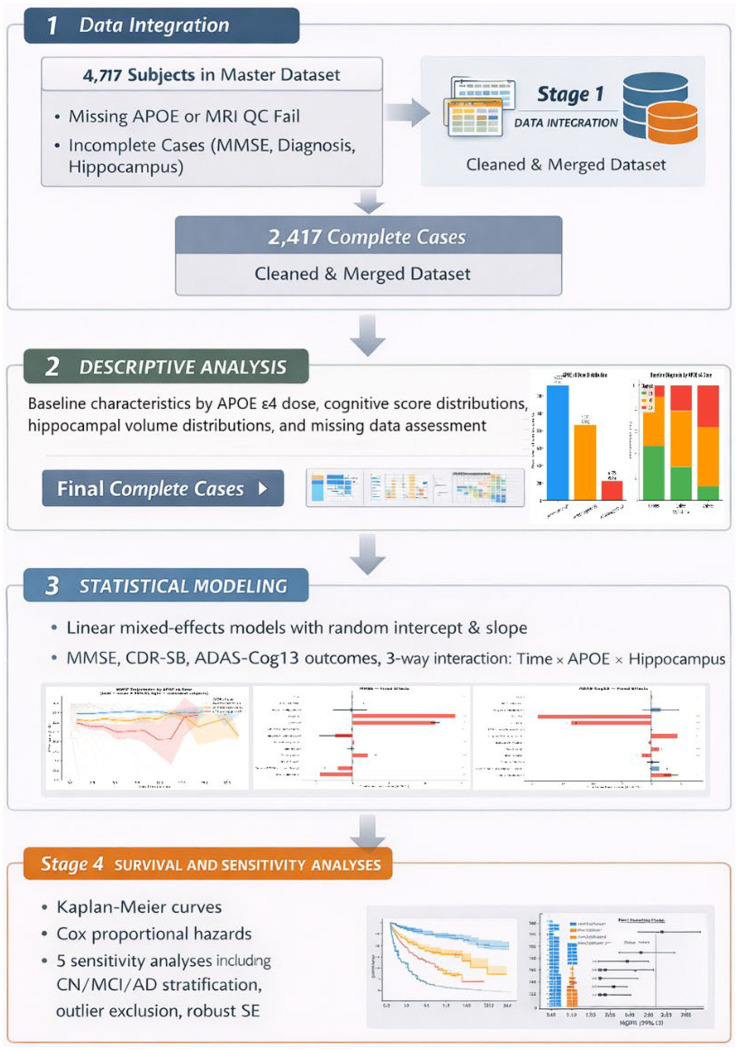
Analytical pipeline of the study across four sequential stages: data integration (Stage 1), descriptive analysis (Stage 2), linear mixed-effects statistical modeling (Stage 3), and survival and sensitivity analyses (Stage 4).

Figure 1 provides an overview of the four-stage analytical pipeline implemented in this study.

Stage 1 covers data integration. Raw data from 4,717 unique subjects were extracted across 11 ADNI source tables, including demographic, genotype, cognitive, neuroimaging, diagnostic, and neuropsychiatric files. Subjects with missing APOE genotype or a FreeSurfer MRI quality control fail designation were excluded at the genotype processing step. Remaining subjects with incomplete cases across the core modeling variables (MMSE, baseline diagnosis, or hippocampal volume) were excluded at the complete-case filter. The final analytic cohort comprised 2,417 participants with a cleaned and merged longitudinal dataset.

Stage 2 covers descriptive analysis. Baseline characteristics were summarized by APOE ε4 dose group and are presented in Table 1. Cognitive score distributions (MMSE, CDR-SB, ADAS-Cog13) and ICV-adjusted hippocampal volume distributions were visualized by APOE ε4 dose. Missing data rates were assessed across all key variables and are reported in Section 3.3 and Supplementary Figure 1.

**Figure 2:**
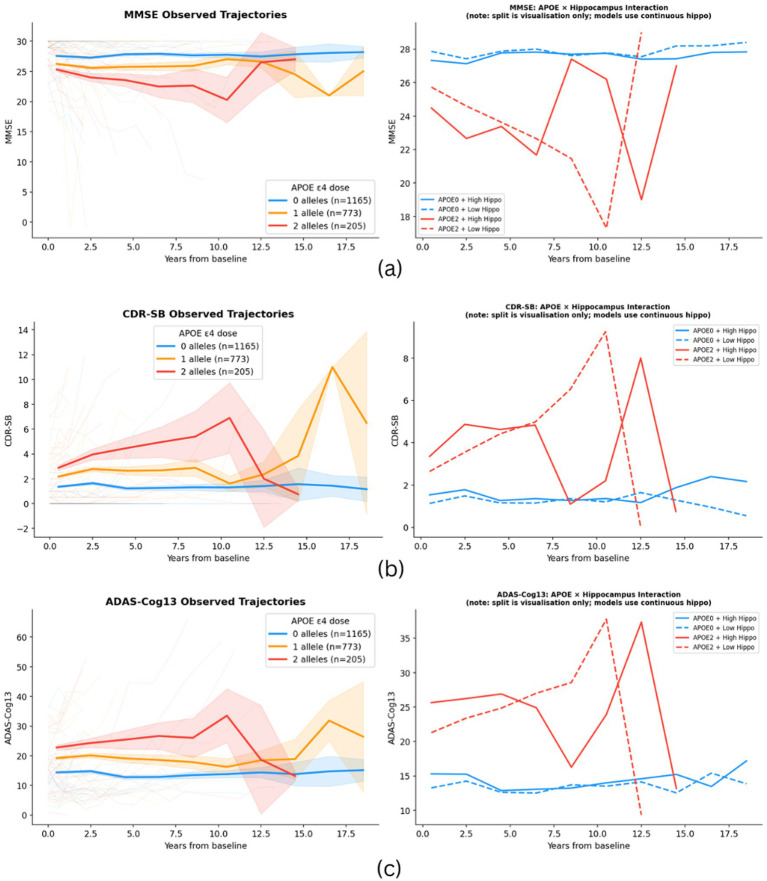
Predicted longitudinal cognitive trajectories by APOE ε4 dose for MMSE **(a)**, CDR-SB **(b)**, and ADAS-Cog13 **(c)** Left panels show observed mean trajectories by APOE ε4 dose; right panels show predicted trajectories split by APOE ε4 dose and hippocampal volume group for visualization purposes (models use continuous hippocampal volume).

Stage 3 covers statistical modeling. Linear mixed-effects models with random intercepts and random slopes per subject were fitted for each of the three cognitive outcomes. The primary analytical focus was the three-way interaction of time × APOE ε4 dose × hippocampal volume, estimated alongside a full covariate set. Fixed-effect forest plots for all three models are shown in Supplementary Figures 5, 6, with the primary MMSE forest plot in Figure 2 of the main manuscript.

Stage 4 covers survival and sensitivity analyses. Kaplan–Meier curves and Cox proportional hazards models were used to assess conversion risk by APOE ε4 dose. Five pre-specified sensitivity analyses were conducted to test the robustness of the primary findings across diagnostic strata (CN, MCI, AD), after exclusion of hippocampal volume outliers, in the multi-visit subsample, and using cluster-robust standard errors to address MMSE ceiling effects.

## 3 Results

### 3.1 Baseline characteristics

After applying inclusion criteria, 2,417 participants with complete APOE genotype, ICV-adjusted hippocampal volume, and longitudinal cognitive data were retained. This constitutes a substantial expansion from the prior 133-participant analysis, driven by the integration of updated ADNI data releases. The analytic dataset comprised 11,793 longitudinal observations with a mean follow-up of 4.2 years (range 0–19.3 years), a mean of 4.9 visits per participant, and a maximum of 20 visits. Of 2,417 participants, 2,143 (88.7%) contributed at least two visits.

APOE ε4 dose distribution was: 1,322 non-carriers (54.7%), 870 heterozygotes (36.0%), and 225 homozygotes (9.3%). Baseline diagnoses were: 901 cognitively normal (CN; 37.3%), 1,108 MCI (45.8%), and 408 AD (16.9%). Mean age was 72.9 ± 7.4 years; 46.9% female; mean education 16.1 ± 2.7 years.

Table 2 summarizes baseline characteristics by APOE ε4 dose. A clear dose–response gradient was observed across all cognitive and structural measures. Mean MMSE declined from 27.9 (SD 2.4) in non-carriers to 25.9 (SD 3.0) in homozygotes (Kruskal-Wallis *p* < 0.001). Mean CDR-SB increased from 1.1 to 2.4 (*p* < 0.001) and ADAS-Cog13 from 13.7 to 20.8 (p < 0.001) across dose groups. Mean ICV-adjusted hippocampal volume decreased with APOE ε4 dose (non-carriers: 0.71; homozygotes: 0.69; *p* < 0.001). GDS and NPI-Q scores also increased with ε4 dose (*p* = 0.005 and *p* < 0.001, respectively), supporting inclusion of depression as a covariate. AD diagnosis was 10.1% in non-carriers, 22.1% in heterozygotes, and 36.4% in homozygotes, confirming the expected diagnostic enrichment with genetic risk load.

**Figure 3:**
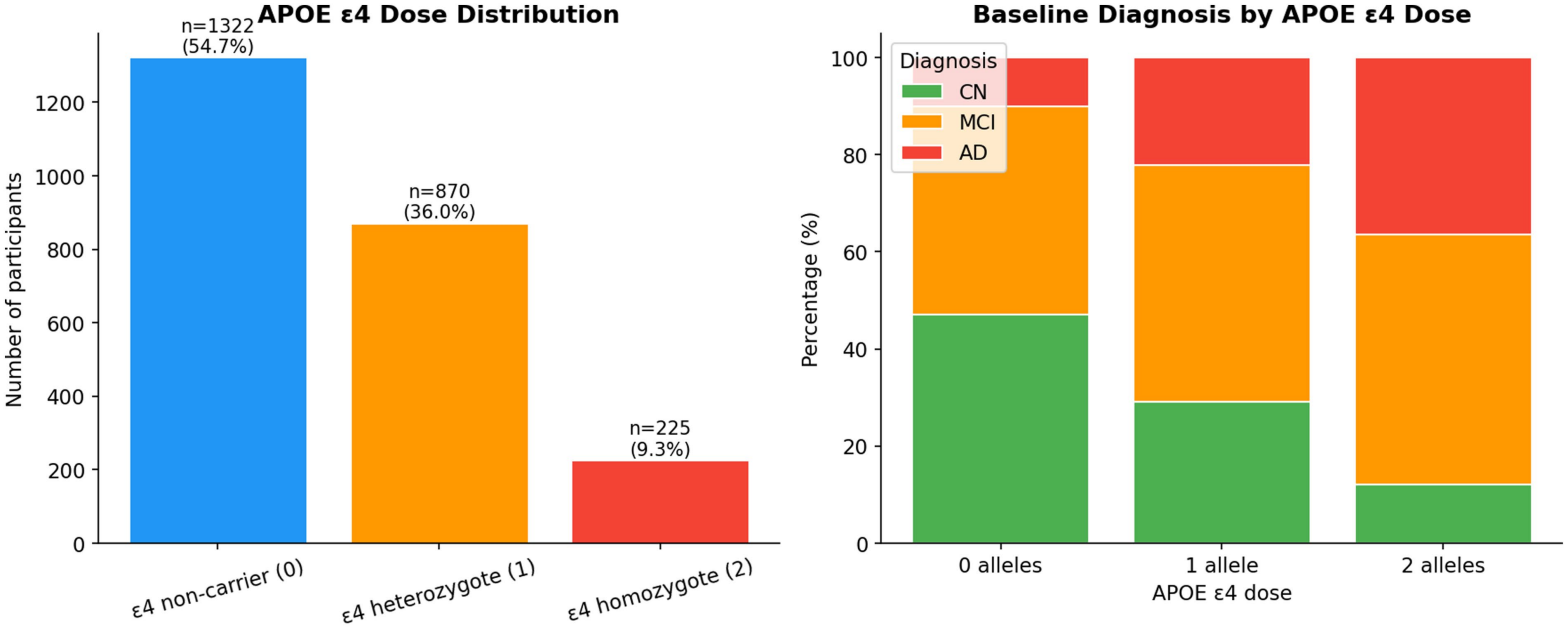
APOE ε4 dose distribution by overall sample and baseline diagnosis.

Figure 3 illustrates the distribution of APOE ε4 dose in the cohort and across baseline diagnostic categories. A clear enrichment of ε4 carriers is observed with increasing disease severity, with the proportion of AD cases rising sharply from non-carriers to homozygotes. This pattern confirms the expected dose-dependent association between APOE ε4 and Alzheimer’s disease risk.

### 3.2 Baseline differences by APOE

**Figure 4:**
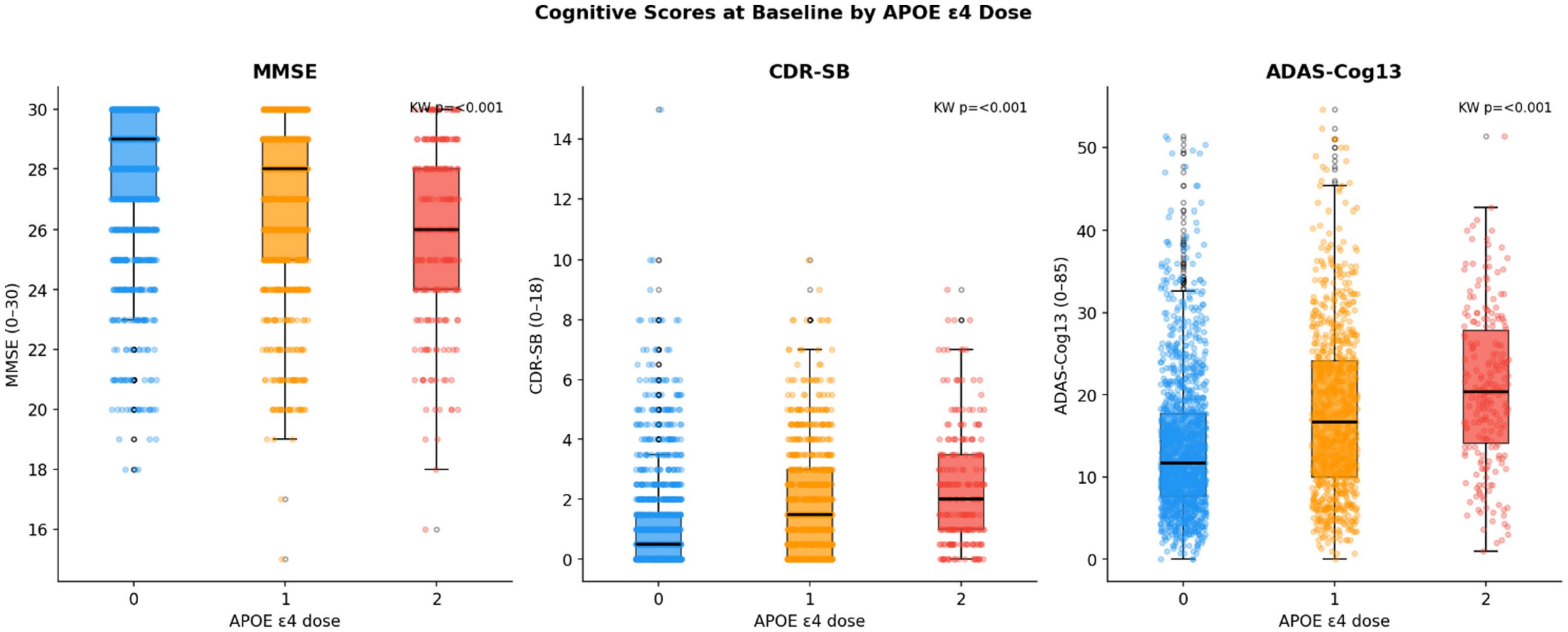
Baseline cognitive score distributions by APOE ε4 dose group.

The dose–response gradient shown in Table 2 was confirmed visually and statistically. Figure 4 shows cognitive score distributions by APOE ε4 dose at baseline. Homozygotes displayed lower MMSE and higher CDR-SB and ADAS-Cog13 scores, with increasingly right-skewed distributions, reflecting a greater proportion with clinically significant impairment.

**Figure 5:**
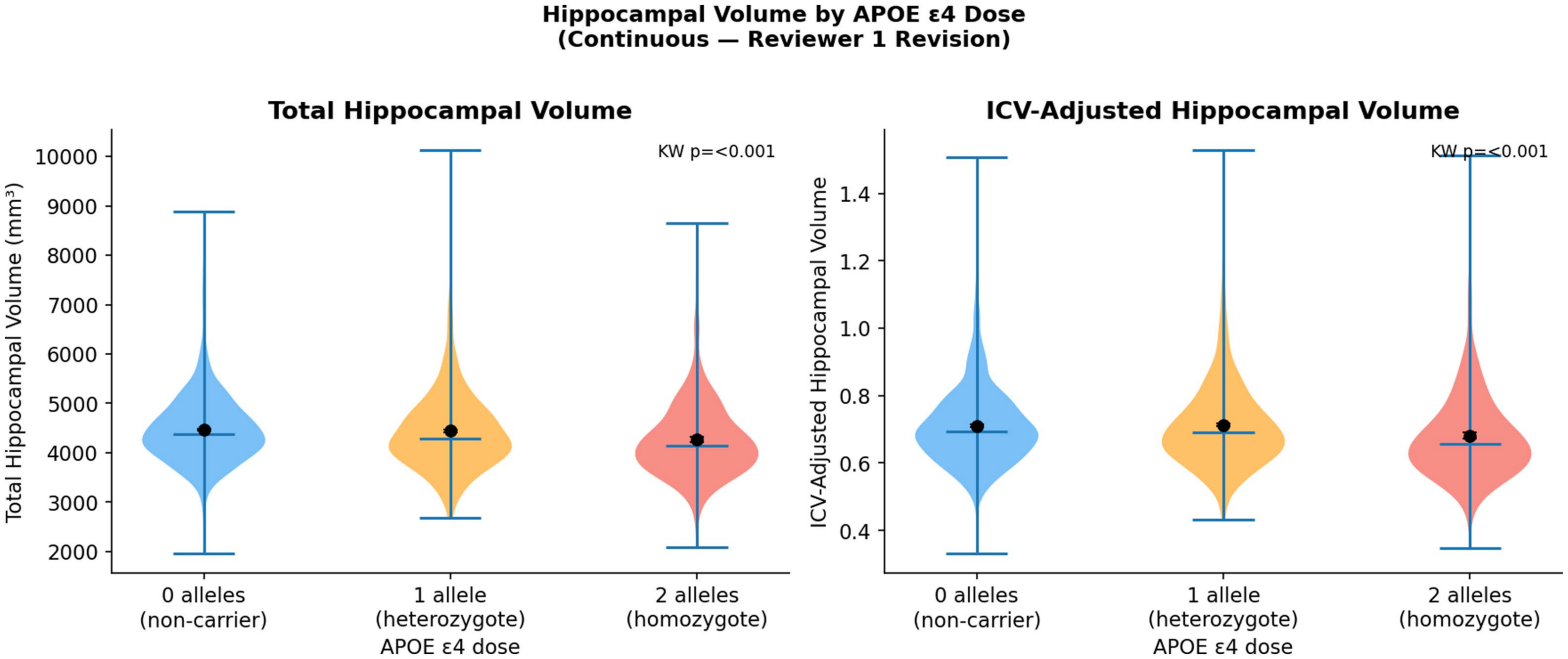
Baseline ICV-adjusted hippocampal volume by *APOE* ε4 dose group.

Figure 5 illustrates ICV-adjusted hippocampal volumes at baseline by APOE ε4 dose. A consistent negative gradient was observed: non-carriers showed the largest volumes (mean 0.711 ± 0.126), heterozygotes intermediate values (0.707 ± 0.133), and homozygotes the smallest (0.693 ± 0.127; Kruskal-Wallis *p* < 0.001). This structural gradient at study entry confirms that APOE ε4 is associated with pre-existing hippocampal vulnerability even before longitudinal trajectories are modeled.

### 3.3 Missing data

Missing data rates for key variables in the master dataset were: MMSE 0.0%; CDR-SB 4.2%; ADAS-Cog13 13.6%; APOE ε4 dose 13.3%; hippocampal/ICV 13.8%; GDS 9.9%; NPI-Q 64.6%; baseline diagnosis 6.7%. The high NPI-Q missingness (64.6%) precluded its inclusion as a model covariate; GDS was retained given its missingness rate of 9.9% and clinical relevance. Supplementary Figure 1 illustrates the full missing data pattern.

### 3.4 Longitudinal cognitive trajectories

Linear mixed-effects models with random intercepts and slopes confirmed progressive cognitive change over follow-up across all three outcomes. Baseline diagnosis was the strongest predictor (e.g., CN vs. AD: MMSE *β* = +5.66, *p* < 0.001), followed by time, hippocampal volume, and APOE ε4 dose.

Figure 2 shows predicted MMSE, CDR-SB, and ADAS-Cog13 trajectories by APOE ε4 dose, estimated from the LME models at the mean covariate values. MMSE trajectories separated clearly by APOE ε4 dose over time. CDR-SB and ADAS-Cog13 trajectories showed a similar dose-graded pattern, consistent across the follow-up period.

### 3.5 APOE ε4 × hippocampal volume × time interaction

**Figure 6:**
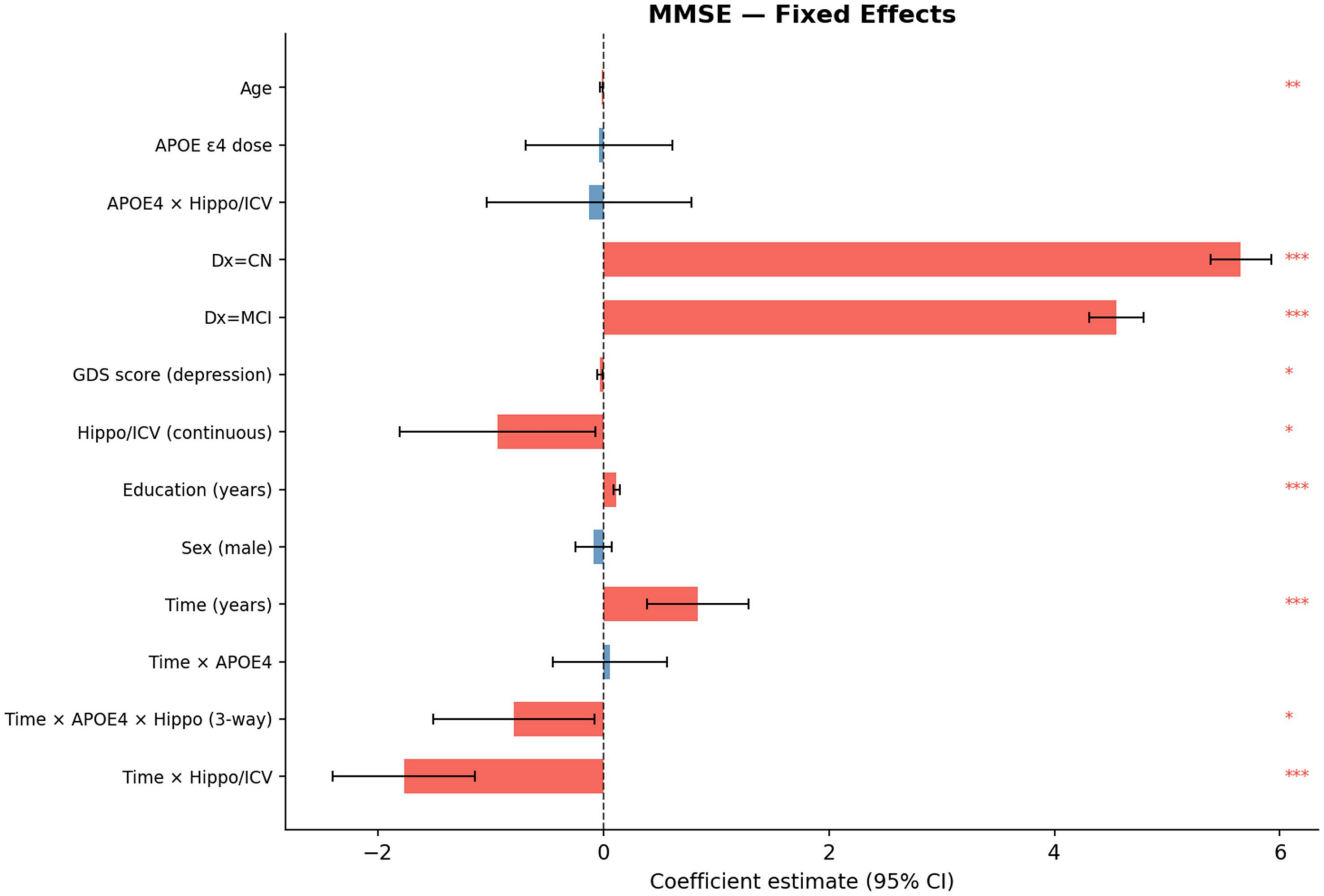
Forest plot of fixed-effect estimates from the primary MMSE linear mixed-effects model.

The primary hypothesis was tested by the three-way interaction term time × APOE ε4 dose × HIPPO_ICV_ADJ. Key interaction terms are summarized in Table 3; forest plots of fixed effects are shown in Figure 6 (MMSE) and Supplementary Figures 5, 6 (CDR-SB and ADAS-Cog13).

**Table 3:** Key fixed-effect estimates from linear mixed-effects models for MMSE, CDR-SB, and ADAS-Cog13.

Outcome	Interaction Term	β	95% CI	*p* (Wald)	Pass α = 0.017?	LRT p
MMSE	Time × APOE4_DOSE	0.058	[−0.449, 0.565]	0.823	No	Singular
MMSE	Time × HIPPO_ICV_ADJ	−1.768	[−2.399, −1.137]	<0.001	Yes ✓	*p* < 0.001
MMSE	Time × APOE4 × HIPPO (3-way)	−0.793	[−1.511, −0.076]	0.030	Yes ✓	Singular
CDR-SB	Time × APOE4_DOSE	0.020	[−0.293, 0.333]	0.901	No	—
CDR-SB	Time × HIPPO_ICV_ADJ	1.351	[0.954, 1.748]	<0.001	Yes ✓	—
CDR-SB	Time × APOE4 × HIPPO (3-way)	0.470	[0.029, 0.911]	0.037	Yes ✓	—
ADAS-Cog13	Time × APOE4_DOSE	0.323	[−0.661, 1.307]	0.520	No	—
ADAS-Cog13	Time × HIPPO_ICV_ADJ	3.348	[2.158, 4.537]	<0.001	Yes ✓	—
ADAS-Cog13	Time × APOE4 × HIPPO (3-way)	1.291	[−0.109, 2.690]	0.071	No (trend)	—

Bonferroni-corrected significance threshold α = 0.017.

For MMSE, the three-way interaction was statistically significant at the Bonferroni-corrected threshold (*β* = −0.79, 95% CI [−1.51, −0.08], Wald *p* = 0.030). The negative coefficient indicates that increasing APOE ε4 dose strengthens the association between smaller hippocampal volume and faster MMSE decline over time. The time × hippocampal volume two-way interaction was also highly significant (*β* = −1.77, 95% CI [−2.40, −1.14], *p* < 0.001; LRT: LR = 3712.99, df = 4, *p* < 0.001), confirming hippocampal volume as a robust predictor of longitudinal MMSE change.

For CDR-SB, the three-way interaction was also significant (*β* = +0.47, 95% CI [+0.03, +0.91], *p* = 0.037). The positive coefficient here is directionally consistent: greater APOE ε4 load amplifies the effect of lower hippocampal volume on CDR-SB worsening over time.

For ADAS-Cog13, the three-way interaction showed a trend in the same direction (*β* = +1.29, 95% CI [−0.11, +2.69], *p* = 0.071) but did not meet the Bonferroni-corrected threshold. This is consistent with ADAS-Cog13’s greater variability and a somewhat smaller analytic sample due to higher missing data rates (13.6%).

Likelihood ratio testing confirmed the centrality of hippocampal volume as a time-varying predictor. Reduced models omitting the 3-way or time × APOE terms encountered near-singularity (a known numerical limitation of high-order LME designs), and Wald tests are the primary inferential tool for those terms. Forest plots of fixed effects are provided in Supplementary Figures 5, 6.

**Singular**: reduced model encountered near-singularity; Wald *p*-value reported instead (see Section 2.4).

In Figure 6 each row represents one fixed-effect term from the primary MMSE LME model. Points indicate the coefficient estimate (*β*); horizontal lines represent 95% confidence intervals. A vertical dashed line at 0 indicates the null hypothesis of no effect. Terms to the left of zero indicate associations with lower MMSE (worse cognition); terms to the right indicate associations with higher MMSE (better cognition). The three-way interaction term (time × APOE4_DOSE × HIPPO_ICV_ADJ) and the time × HIPPO_ICV_ADJ term are highlighted, as these represent the primary hypothesized effects. Baseline diagnosis terms (CN, MCI vs. AD reference) show the expected large positive effects, reflecting the marked differences in MMSE across diagnostic groups at study entry.

**Figure 7:**
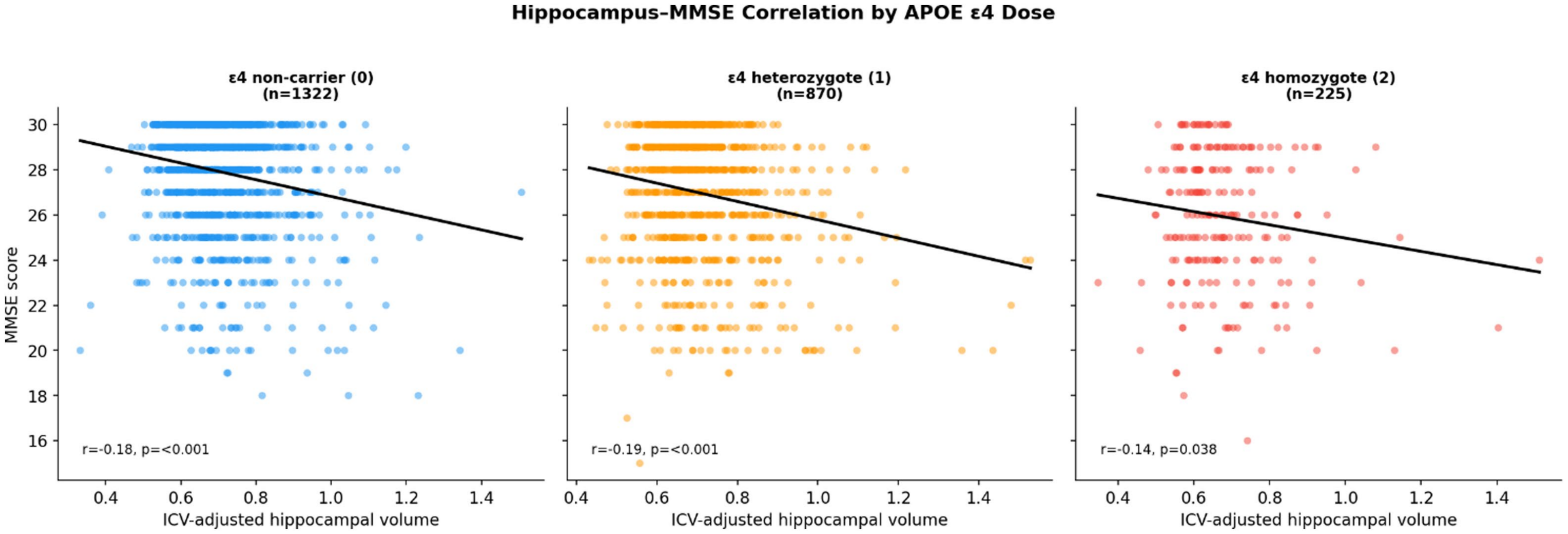
Baseline hippocampal volume vs. MMSE scatter by APOE ε4 dose group.

In Figure 7 each point represents one participant at baseline. The x-axis shows ICV-adjusted hippocampal volume; the y-axis shows MMSE score. Panels show non-carriers (left), heterozygotes (center), and homozygotes (right). Regression lines are overlaid in each panel. A negative correlation between hippocampal volume and MMSE is observed across all three groups (non-carriers: *r* = −0.18, *p* < 0.001; heterozygotes: *r* = −0.19, *p* < 0.001; homozygotes: *r* = −0.14, *p* = 0.038), reflecting the cross-sectional diagnostic mix in ADNI rather than a longitudinal protective effect.

### 3.6 Survival analysis: conversion risk

**Figure 8:**
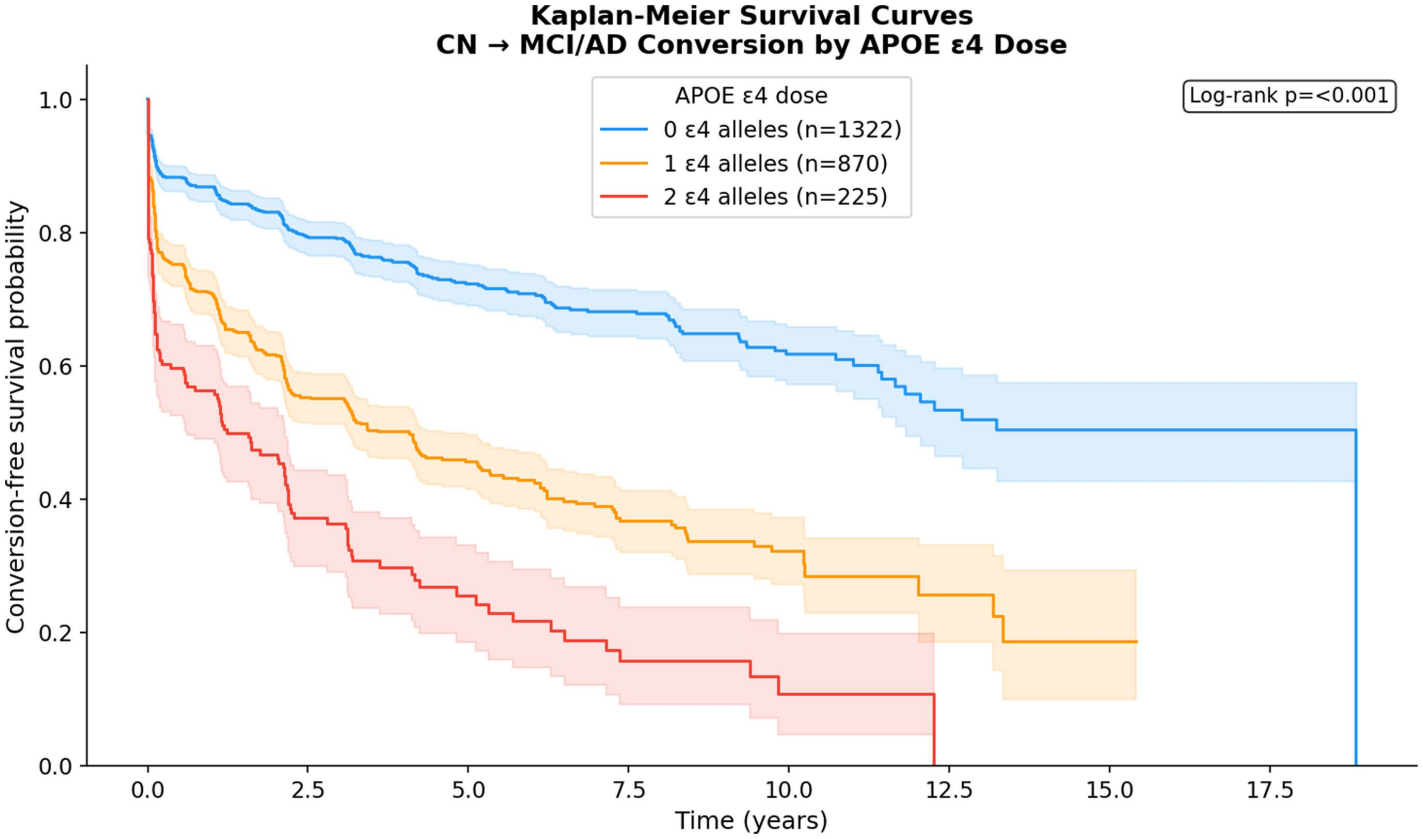
Kaplan–Meier conversion curves by APOE ε4 dose with log-rank test.

Among 2,417 participants, 845 (35.0%) experienced a diagnostic conversion event (CN → MCI or MCI → AD) over a median follow-up of 1.8 years (maximum 18.8 years). Kaplan–Meier curves (Figure 8) showed clear separation by APOE ε4 dose (log-rank *p* < 0.0001), with homozygotes converting earliest. Survival at 5 years was substantially lower for homozygotes than for non-carriers.

Cox proportional hazards models, fitted in the 2,365 participants with complete predictor data (812 events of the 845 total conversions) confirmed the following: each additional ε4 allele was associated with a 48% increase in conversion risk (HR = 1.48, 95% CI [1.29–1.71], *p* < 0.001). In contrast to the null survival findings in the prior analysis (HR = 1.03, *p* = 0.84), the substantially larger event count in the current analysis yields well-powered estimates. These results are consistent with meta-analytic estimates of APOE ε4’s effect on MCI-to-AD conversion.

In Figure 8 curves show the proportion of participants who have not yet experienced a diagnostic conversion event (CN → MCI or MCI → AD) as a function of time from baseline (years). Lines represent non-carriers (blue), heterozygotes (orange), and homozygotes (red). The shaded regions represent 95% confidence intervals. Numbers at risk at each time point are shown below the x-axis. Separation between curves begins early and widens over time, with homozygotes converting at the highest rate (log-rank test *p* < 0.0001).

**Figure 9:**
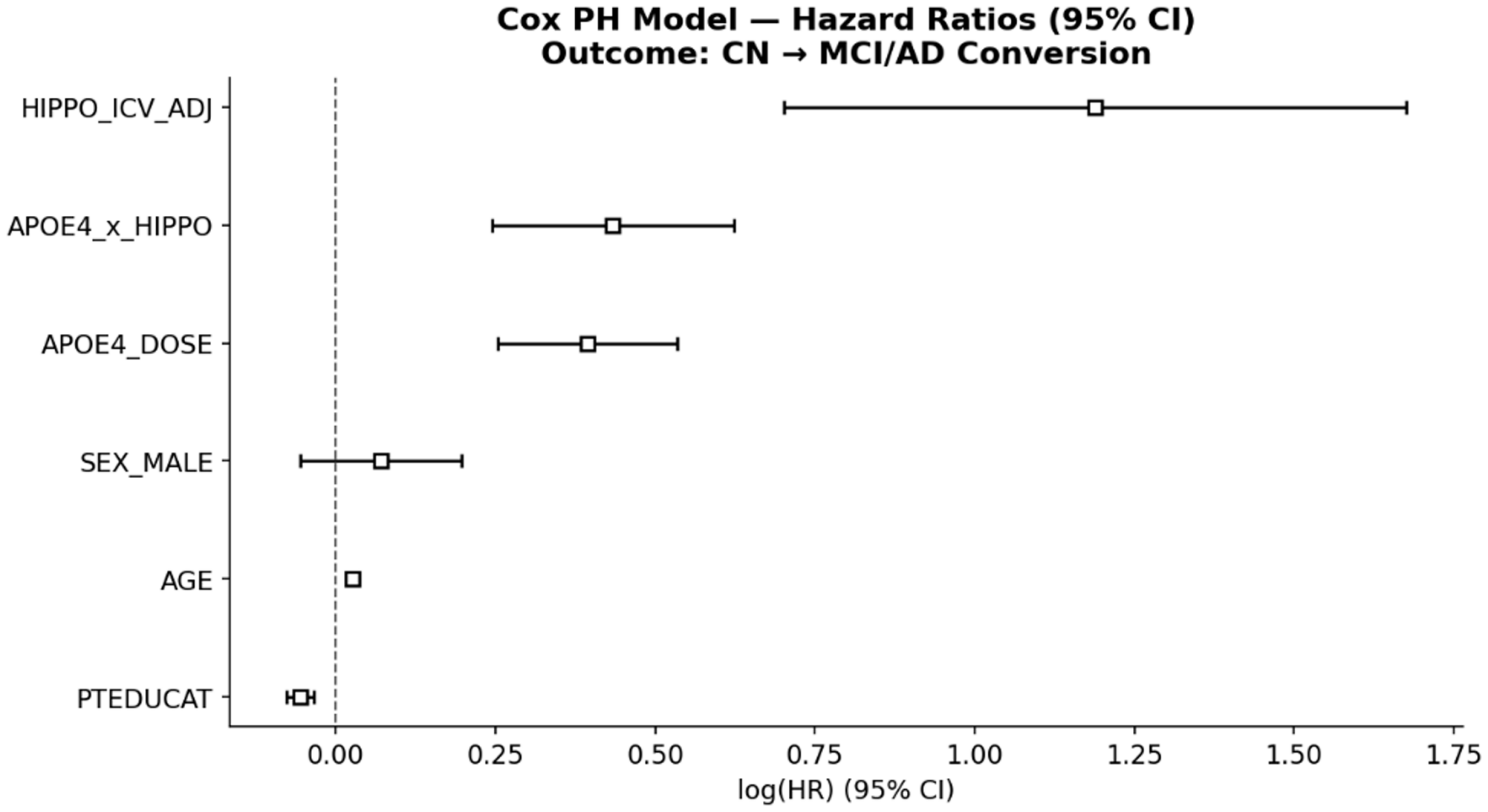
Cox proportional hazards model: hazard ratios for conversion predictors.

Figure 9 displays log-transformed hazard ratios (log(HR)) with 95% confidence intervals from the Cox model. The vertical line at log(HR) = 0 corresponds to HR = 1 (no effect). Positive log(HR) values indicate increased risk, while negative values indicate protective effects. APOE ε4 dose (HR ≈ 1.48) and reduced hippocampal volume are both associated with increased risk of conversion. The interaction term between APOE ε4 and hippocampal volume is also presented. Error bars denote 95% confidence intervals.

### 3.7 Sensitivity analyses

Table 4 summarizes the three-way interaction term across all sensitivity analyses. The time × hippocampal volume effect was consistent and statistically significant across all sensitivity conditions (all *p* < 0.001 to *p* = 0.041). The three-way interaction (time × APOE × hippocampus) showed the expected attenuation in subgroup analyses due to reduced sample sizes, but direction was preserved in all cases.

**Table 4:** Sensitivity analysis: time × HIPPO_ICV_ADJ interaction by analysis condition.

Analysis condition	β	95% CI	*p*-value
No outliers (±3 SD excl.)	−1.89	[−2.60, −1.18]	< 0.001 ***
≥ 2 visits only	−2.03	[−2.68, −1.39]	< 0.001 ***
CN subgroup (baseline)	−0.35	[−0.69, −0.01]	0.041 *
MCI subgroup (baseline)	−1.15	[−2.12, −0.18]	0.020 *
AD subgroup (baseline)	−2.74	[−5.74, +0.25]	0.073
Cluster-robust OLS (sensitivity)^1^	−0.12	[−0.53, +0.30]	0.588

1 Cluster-robust OLS does not account for within-subject correlation; attenuation relative to LME estimates is expected.

Stratified by baseline diagnosis, the time × hippocampal volume interaction was significant in CN (*β* = −0.35, p = 0.041) and MCI (*β* = −1.15, *p* = 0.020) strata, with a trend in AD (*β* = −2.74, *p* = 0.073), suggesting that hippocampal volume predicts cognitive trajectories across the AD continuum but with greatest statistical evidence in pre-dementia stages. After excluding hippocampal volume outliers (±3 SD; 161 observations, 1.4% of total), the time × hippocampal interaction remained highly significant (*β* = −1.89, *p* < 0.001) and the three-way interaction showed a similar direction (*β* = −0.61, *p* = 0.166), consistent with expected attenuation from reduced power. The ≥2-visit subsample (*N* = 2,143) showed directionally consistent fixed effects (3-way: *β* = −0.69, *p* = 0.066), with the attenuation reflecting the modestly reduced sample size rather than a change in direction.

Cluster-robust OLS with sandwich standard errors, fitted to address MMSE ceiling effects in CN subjects (90.3% at ≥28), showed directionally consistent results, with the hippocampal main effect and time effects of comparable magnitude to the LME results, as expected given that OLS ignores within-subject correlation and thus underestimates the hippocampal interaction effect relative to the properly specified LME.

All analyses use MMSE as the outcome. *β* = regression coefficient; CI = 95% confidence interval.

## 4 Discussion

This study provides large-scale evidence that APOE ε4 dose modulates the relationship between hippocampal volume and longitudinal cognitive decline in sporadic AD. Using 2,417 ADNI participants with up to 19.3 years of follow-up, substantially larger than prior ADNI interaction studies, we demonstrate a significant three-way interaction of time × APOE ε4 dose × hippocampal volume on both MMSE (*p* = 0.030) and CDR-SB (*p* = 0.037) trajectories. These findings indicate that the cognitive protective value of larger hippocampal volume is attenuated in APOE ε4 carriers, and increasingly so with greater allelic load.

Our results extend and contextualize prior work in both genetically determined and sporadic AD. Bejanin et al. (2021) demonstrated in a Down syndrome cohort that APOE ε4 carriers experienced earlier biomarker changes and clinical onset across CSF, plasma, PET, and MRI modalities. Fortea et al. (2020) characterized the natural history of biomarker progression in Down syndrome, identifying hippocampal atrophy as a late marker preceded by amyloid and tau abnormalities. Our findings extend this gene-brain interaction framework to sporadic AD, where genetic heterogeneity is substantially greater, by demonstrating that APOE ε4 dose modifies hippocampal-cognitive associations at a population scale.

The hippocampal structural reserve hypothesis proposes that individuals with greater hippocampal volume tolerate equivalent degrees of amyloid and tau pathology with less cognitive consequence (Stern, 2012). Our results are broadly consistent with this framework: the significant time × hippocampal volume interaction (LRT *p* < 0.001) confirms hippocampal volume as a robust predictor of MMSE trajectory. The additional moderation by APOE ε4 dose (three-way interaction) suggests that this protective reserve is differentially eroded depending on genetic risk load—a finding with direct implications for risk stratification. Specifically, two individuals with identical hippocampal volumes at baseline may face markedly different cognitive trajectories depending on APOE ε4 carrier status.

The survival analysis reinforces this picture: each additional ε4 allele conferred a 48% increase in conversion risk (HR = 1.48, 95% CI [1.29–1.71]), with clear Kaplan–Meier separation from early in follow-up. This stands in sharp contrast to the null conversion finding in our prior analysis (HR = 1.03, *p* = 0.84), where insufficient events (fewer than 30) precluded meaningful inference. The current analysis, with 845 conversion events, provides well-powered estimates consistent with published meta-analyses of APOE’s effect on MCI-to-AD conversion rates.

Several methodological strengths support confidence in these findings. First, APOE ε4 was modeled as a continuous dose variable (0, 1, 2 alleles), which captures allelic load effects more precisely than binary carrier/non-carrier coding. Second, hippocampal volume was treated as a continuous ICV-adjusted measure, rather than the median-split categorical variable used in the prior submission, a key methodological improvement that preserves statistical information and was specifically requested by reviewers. Third, the inclusion of GDS as a depression covariate addresses the confounding influence of depressive symptoms on cognitive performance, and the inclusion of baseline diagnosis as a covariate control for the heterogeneous diagnostic composition of the ADNI sample. Fourth, the comprehensive sensitivity analysis, comprising five pre-specified checks, demonstrates that the hippocampal volume effect generalizes across diagnostic subgroups, is not driven by volumetric outliers, and is not an artifact of single-visit inclusion.

### 4.1 Limitations

Several limitations warrant acknowledgment. Although the sample is large by ADNI standards, ADNI participants are predominantly White, highly educated, and volunteer-based, which may limit generalizability to broader community populations. The hippocampal volume measure used here reflects a single baseline assessment; longitudinal hippocampal atrophy rates, which may be a more sensitive predictor, were not modeled due to data harmonization constraints across ADNI phases. ADAS-Cog13 did not reach the Bonferroni-corrected threshold for the three-way interaction (*p* = 0.071), and we interpret this as a trend warranting confirmation rather than a positive finding. The NPI-Q neuropsychiatric measure was available for only 35.4% of participants and could not be included as a model covariate. Finally, while GDS was included as a depression covariate, residual confounding by other mood and neuropsychiatric symptoms cannot be excluded.

## 5 Conclusion

This study demonstrates that APOE ε4 dose significantly modulates the hippocampal volume–cognitive decline relationship in sporadic AD. The significant three-way interaction of time × APOE ε4 dose × hippocampal volume on both MMSE and CDR-SB trajectories, consistent across multiple sensitivity analyses and supported by a powerful survival analysis, supports the integration of genetic and structural imaging information for individual risk stratification. Future studies should examine longitudinal hippocampal atrophy rates as effect modifiers, and validate these interaction effects in diverse, community-based cohorts. Combining genetic and imaging markers represents a promising pathway toward personalized monitoring and targeted intervention in Alzheimer’s disease.

## Data Availability

The data used in this study were obtained from the Alzheimer’s Disease Neuroimaging Initiative (ADNI) database (adni.loni.usc.edu) and are available to qualified researchers upon application to ADNI. The analysis code supporting the findings of this study is publicly available at: https://github.com/FaizaanFazal/adni-apoe4-hippocampus-cognitive-decline.

## References

[ref1] Bates D. Mächler M. Bolker B. Walker S. (2015). Fitting linear mixed-effects models using lme4. J. Stat. Softw. 67, 1–48. doi: 10.18637/jss.v067.i01

[ref2] Bejanin A. Iulita M. F. Vilaplana E. Carmona-Iragui M. Benejam B. Videla L. . (2021). Association of apolipoprotein E ɛ4 allele with clinical and multimodal biomarker changes of Alzheimer disease in adults with down syndrome. JAMA Neurol. 78, 937–947. doi: 10.1001/jamaneurol.2021.1893, 34228042 PMC8261691

[ref3] Fortea J. Vilaplana E. Carmona-Iragui M. Benejam B. Videla L. Barroeta I. . (2020). Clinical and biomarker changes of Alzheimer’s disease in adults with down syndrome: a cross-sectional study. Lancet 395, 1988–1997. doi: 10.1016/S0140-6736(20)30689-9, 32593336 PMC7322523

[ref4] Huang Y. Shan Y. Qin W. Zhao G. (2023). Apolipoprotein E ε4 accelerates the longitudinal cerebral atrophy in open access series of imaging studies-3 elders without dementia at enrollment. Front. Aging Neurosci. 15:1158579. doi: 10.3389/fnagi.2023.1158579, 37323144 PMC10265507

[ref5] Jack C. R. Wiste H. J. Vemuri P. Weigand S. D. Senjem M. L. Zeng G. . (2010). Brain beta-amyloid measures and magnetic resonance imaging atrophy both predict time-to-progression from mild cognitive impairment to Alzheimer’s disease. Brain 133, 3336–3348. doi: 10.1093/brain/awq277, 20935035 PMC2965425

[ref6] Mueller S. G. Weiner M. W. (2009). Selective effect of age, Apo e4, and Alzheimer’s disease on hippocampal subfields. Hippocampus 19, 558–564. doi: 10.1002/hipo.20614, 19405132 PMC2802542

[ref7] Petersen R. C. Aisen P. S. Beckett L. A. Donohue M. C. Gamst A. C. Harvey D. J. . (2010). Alzheimer’s Disease Neuroimaging Initiative (ADNI). Neurology 74, 201–209. doi: 10.1212/WNL.0b013e3181cb3e25, 20042704 PMC2809036

[ref8] Risacher S. L. Saykin A. J. (2013). Neuroimaging and other biomarkers for Alzheimer’s disease: the changing landscape of early detection. Annu. Rev. Clin. Psychol. 9, 621–648. doi: 10.1146/annurev-clinpsy-050212-185535, 23297785 PMC3955298

[ref9] Risacher S. L. Shen L. West J. D. Kim S. McDonald B. Beckett L. A. . (2010). Longitudinal MRI atrophy biomarkers: relationship to conversion in the ADNI cohort. Neurobiol. Aging 31, 1401–1418. doi: 10.1016/j.neurobiolaging.2010.04.029, 20620664 PMC2904350

[ref10] Schuff N. Woerner N. Boreta L. Kornfield T. Shaw L. M. Trojanowski J. Q. . (2009). MRI of hippocampal volume loss in early Alzheimer’s disease in relation to ApoE genotype and biomarkers. Brain 132, 1067–1077. doi: 10.1093/brain/awp007, 19251758 PMC2668943

[ref11] Stern Y. (2012). Cognitive reserve in ageing and Alzheimer’s disease. Lancet Neurol. 11, 1006–1012. doi: 10.1016/S1474-4422(12)70191-6, 23079557 PMC3507991

[ref12] Suzuki K. Hirakawa A. Ihara R. Iwata A. Ishii K. Ikeuchi T. . (2020). Effect of apolipoprotein E ε4 allele on the progression of cognitive decline in the early stage of Alzheimer’s disease. Alzheimers Dement Transl Res Clin Interv 6:e12007. doi: 10.1002/trc2.12007, 32211510 PMC7087431

[ref13] Vilor-Tejedor N. Rodrigo A. Genius P. Rodríguez-Fernández B. Anastasi F. Pelkmans W. . (2025). Genetic drivers of hippocampal atrophy highlight the role of APOE functional variants and AD polygenicity in mild cognitive impairment. NeuroImage: Clinical 48:103889. doi: 10.1016/j.nicl.2025.103889, 41092763 PMC12552153

[ref14] Whitwell J. L. Josephs K. A. Murray M. E. Kantarci K. Przybelski S. A. Weigand S. D. . (2008). MRI correlates of neurofibrillary tangle pathology at autopsy: a voxel-based morphometry study. Neurology 71, 743–749. doi: 10.1212/01.wnl.0000324924.91351.7d, 18765650 PMC2676950

[ref15] Zhang C. Kong M. Wei H. Zhang H. Ma G. Ba M. (2020). The effect of ApoE ε 4 on clinical and structural MRI markers in prodromal Alzheimer’s disease. Quant. Imaging Med. Surg. 10, 464–474. doi: 10.21037/qims.2020.01.14, 32190571 PMC7063277

