# Supplementary material for: APOE ε4 as a predictor of cognitive decline and its interaction with hippocampal volume in Alzheimer’s disease
Source: Front Aging Neurosci. 2026 Apr 22;18:1730265. doi: 10.3389/fnagi.2026.1730265 (PMC13144130; doi:10.3389/fnagi.2026.1730265)
Supplement: Supplementary file 1 [file Data_Sheet_1.DOCX]

Supplementary Material

# Supplementary Data

# Supplementary Figures and Tables

## Supplementary Figures


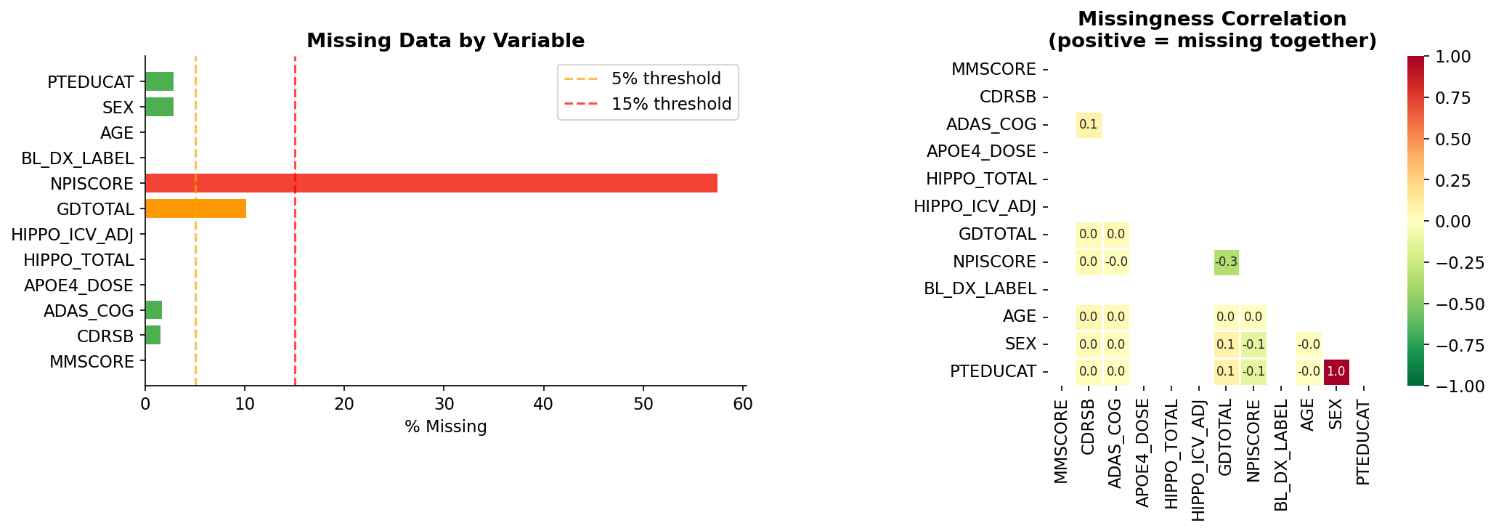


Supplementary Figure 1 Missing data rates and patterns across key variables in the master dataset.


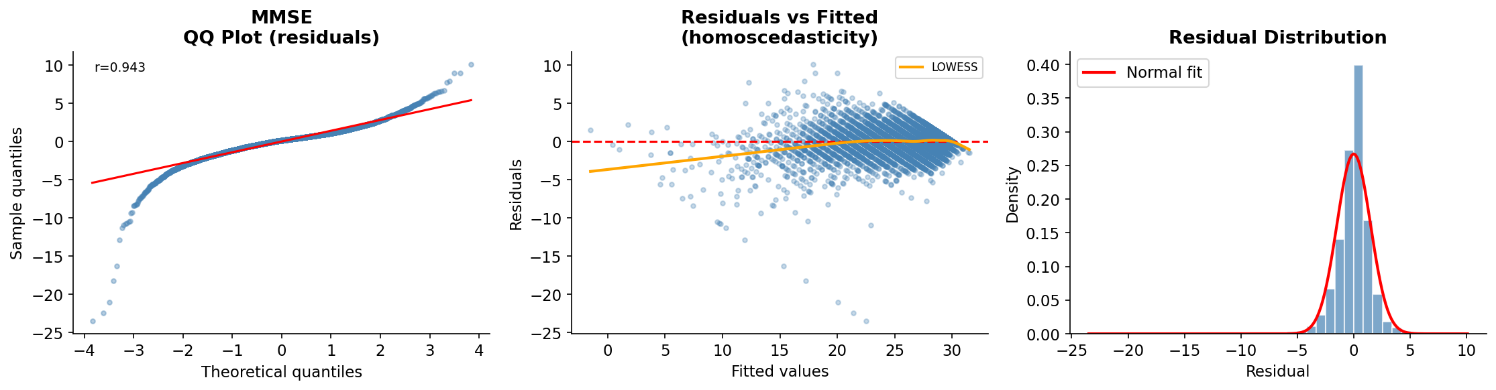


Supplementary Figure 2 MMSE model residual diagnostics: residual-vs-fitted, Q-Q plot, and histogram.


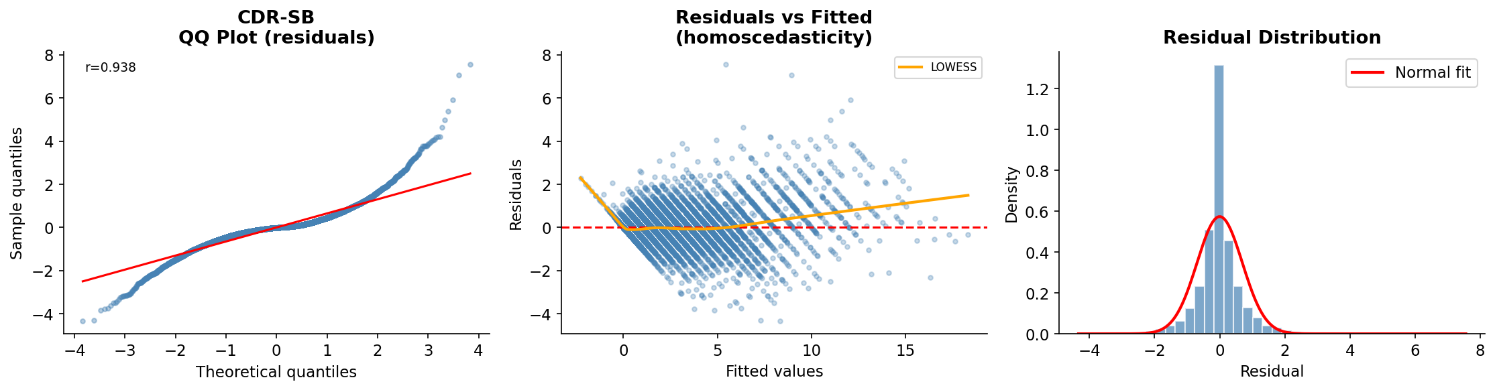


Supplementary Figure 3 CDR-SB model residual diagnostics.


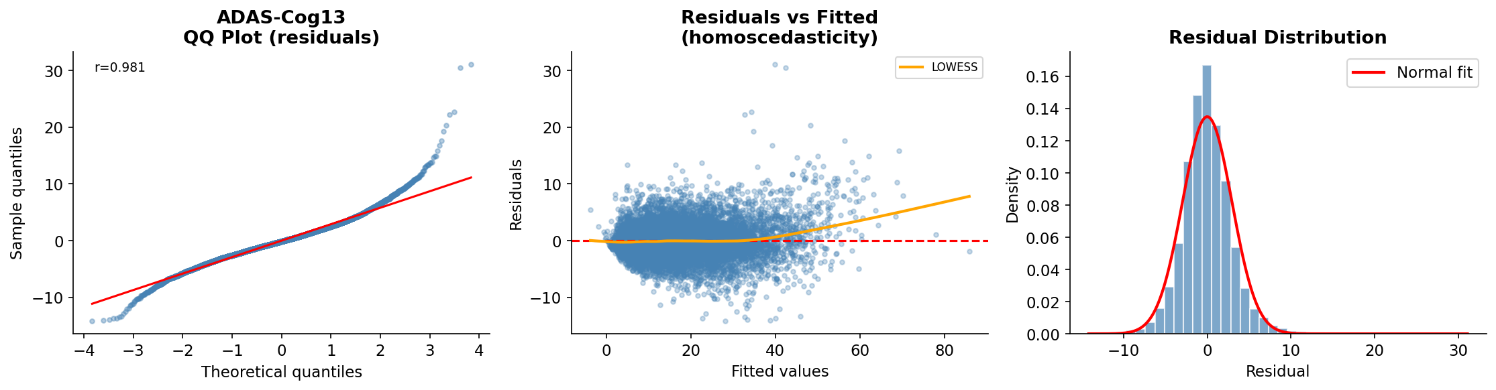


Supplementary Figure 4 ADAS-Cog13 model residual diagnostics.


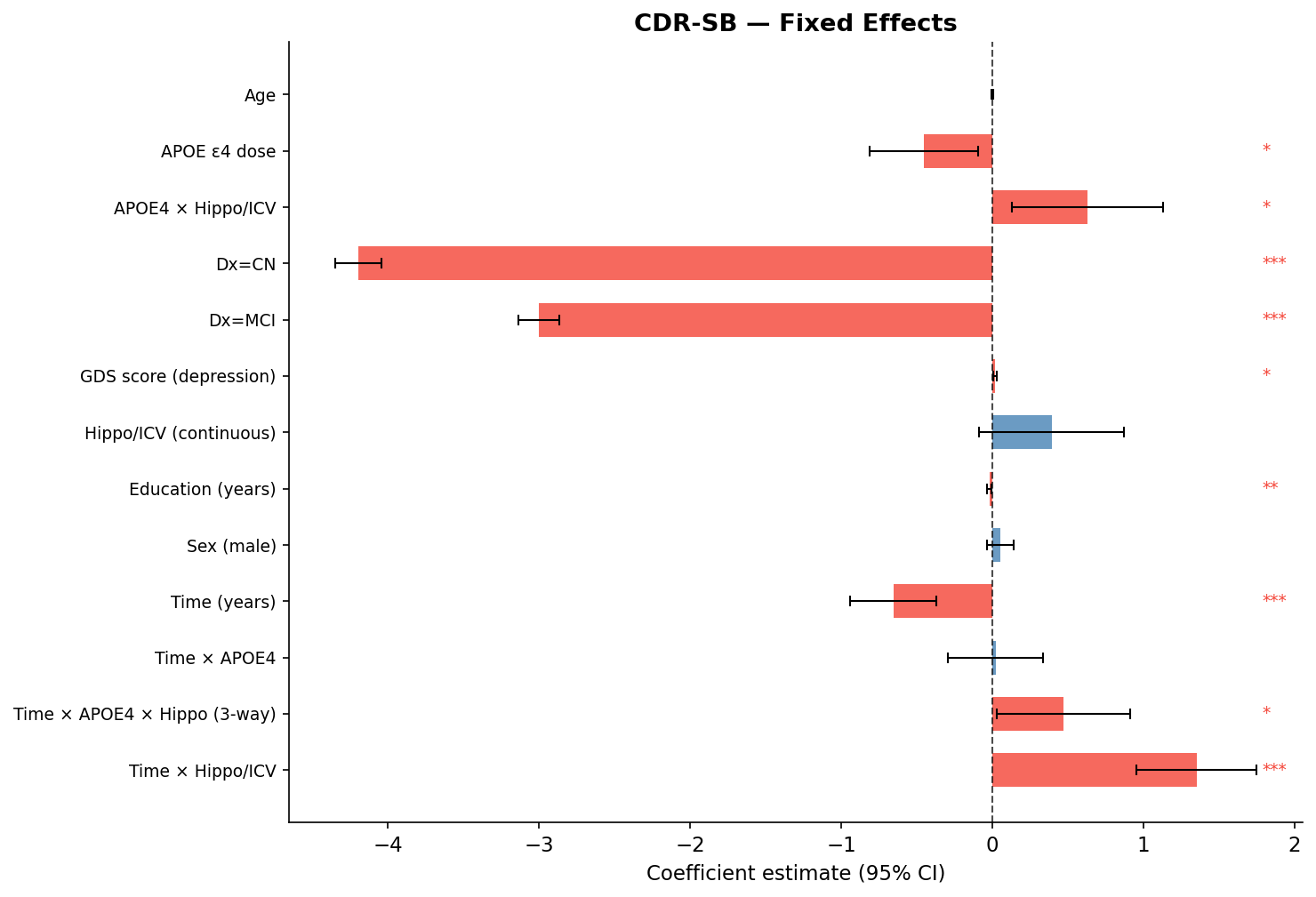


Supplementary Figure 5 Forest plot of full fixed-effect estimates from the CDR-SB model.


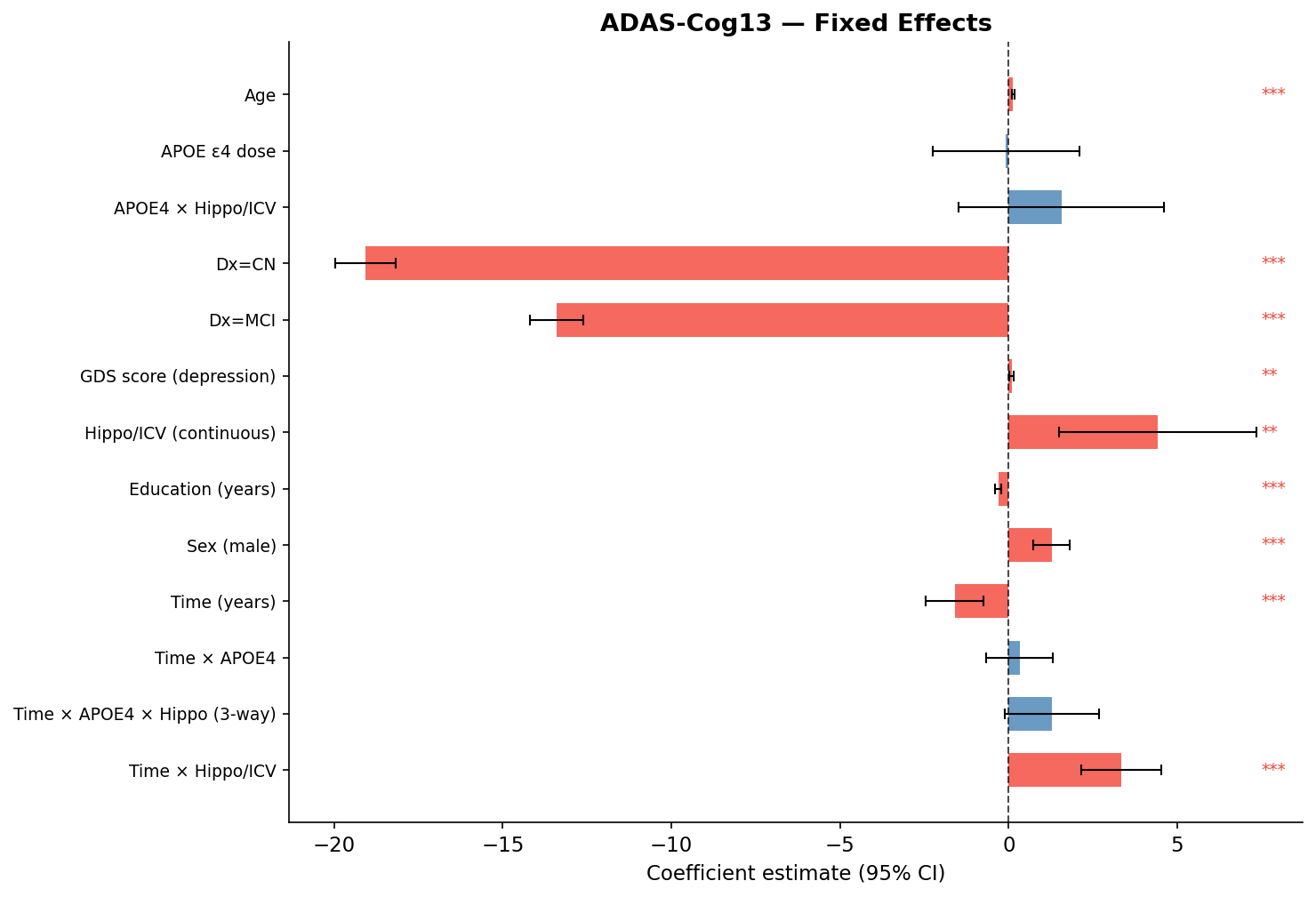


Supplementary Figure 6 Forest plot of full fixed-effect estimates from the ADAS-Cog13 model.


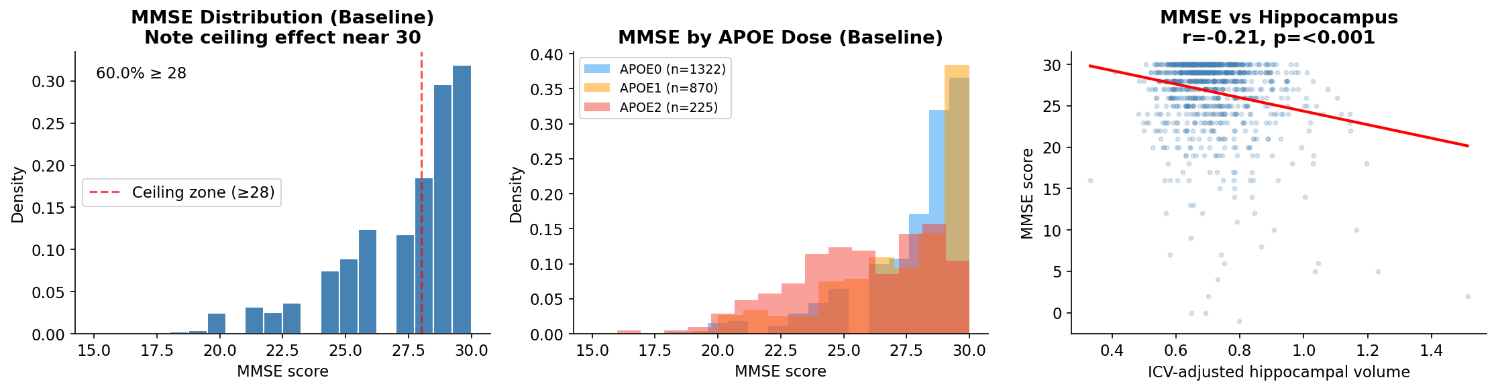


Supplementary Figure 7 MMSE score distribution by baseline diagnosis illustrating ceiling effects.


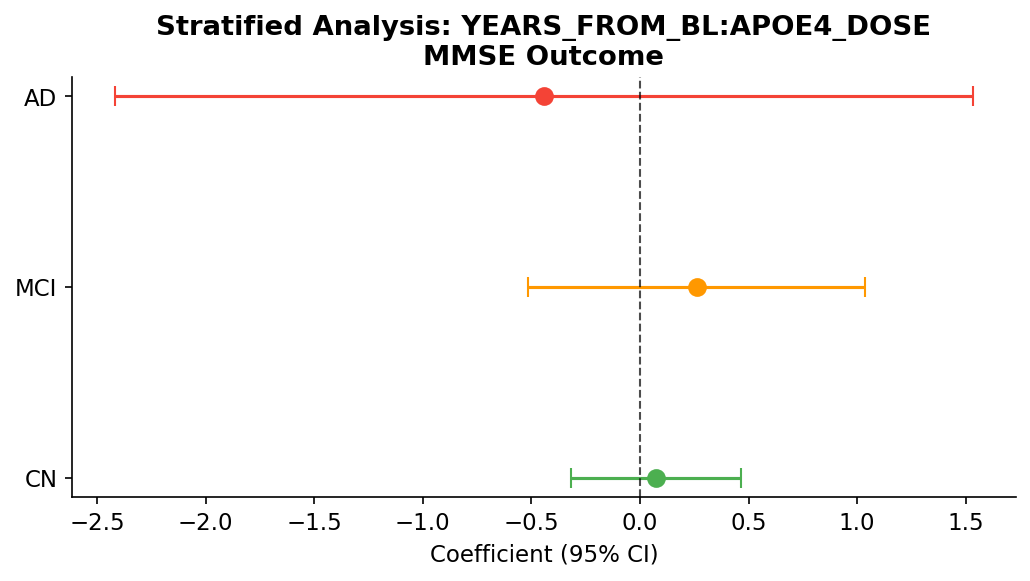


Supplementary Figure 8 Predicted MMSE trajectories by APOE ε4 dose within each baseline diagnosis subgroup.
